# Mysterious abrupt carbon-14 increase in coral contributed by a comet

**DOI:** 10.1038/srep03728

**Published:** 2014-01-16

**Authors:** Yi Liu, Zhao-feng Zhang, Zi-cheng Peng, Ming-xing Ling, Chuan-Chou Shen, Wei-guo Liu, Xiao-chun Sun, Cheng-de Shen, Ke-xin Liu, Weidong Sun

**Affiliations:** 1 CAS Key Laboratory of Crust-Mantle Materials and Environments, School of Earth and Space Sciences, University of Science and Technology of China, Hefei 230026, China; 2 State Key Laboratory of Isotope Geochemistry, Guangzhou Institute of Geochemistry, The Chinese Academy of Sciences, Guangzhou 510640, China; 3 High-Precision Mass Spectrometry and Environment Change Laboratory (HISPEC), Department of Geosciences, National Taiwan University, Taipei 10617, Taiwan; 4 State Key Laboratory of Loess and Quaternary Geology, Institute of Earth Environment, The Chinese Academy of Sciences, Xi'an 710075, China; 5 Institute for the History of Natural Sciences, The Chinese Academy of Sciences, Beijing 100190, China; 6 State Key Laboratory of Nuclear Physics and Technology & Institute of Heavy Ion Physics, School of Physics, Peking University, Beijing 100871, China; 7 CAS Key Laboratory of Mineralogy and Metallogeny, Guangzhou Institute of Geochemistry, The Chinese Academy of Sciences, Guangzhou 510640, China; 8 These authors contributed equally to this work.

## Abstract

A large and sudden increase in radiocarbon (^14^C) around AD 773 are documented in coral skeletons from the South China Sea. The ^14^C increased by ~ 15‰ during winter, and remain elevated for more than 4 months, then increased and dropped down within two months, forming a spike of 45‰ high in late spring, followed by two smaller spikes. The ^14^C anomalies coincide with an historic comet collision with the Earth's atmosphere on 17 January AD 773. Comas are known to have percent-levels of nitrogen by weight, and are exposed to cosmic radiation in space. Hence they may be expected to contain highly elevated ^14^C/^12^C ratios, as compared to the Earth's atmosphere. The significant input of ^14^C by comets may have contributed to the fluctuation of ^14^C in the atmosphere throughout the Earth's history, which should be considered carefully to better constrain the cosmic ray fluctuation.

Carbon-14 (^14^C) is a cosmogenic isotope of C formed on Earth primarily through radiation of atmospheric nitrogen by the reaction:^14^N(n,p)^14^C (refs. 1–4). Its abundance in the atmosphere varies with time^5^, which is generally attributed to variations in the earth's magnetic field, solar activity and changes in the carbon cycle^6^. A large and sudden increase in ^14^C of ~12‰ was reported from a tree ring study in Japan to have occurred between AD 774 and AD 775 (hereafter M12)^7^. Their modeling showed that the atmospheric level of ^14^C must have jumped over the course of no longer than a year, corresponding to an increase 10 times larger than the average production from Galactic cosmic rays and 20 times larger than that expected over 2 × 11 yr solar cycles. The measured values were shown to be too large for a solar flare or local supernova. Given that no detectable increase in ^14^C corresponding to supernovas SN 1006 and SN 1054 were observed^7^,^8^, it is argued that much higher energies would be required for the M12 event, if it is related to a supernova^7^. Alternative explanations for this mysterious ^14^C elevation include a highly energetic radiation burst, e.g., proton storms from giant solar flares^9^,^10^, a giant cometary impact upon the Sun^11^, or floods of γ-rays from supernova explosions^12^. Such high levels of radiation however, might also cause mass extinctions^13^, which are absent following the M12 event. Moreover, it has been argued, based on historical records, that no superflares have occurred in the Sun during the last two millennia^14^.

A simulated carbon cycle model^10^ suggested that the strength of the M12 event was significantly overestimated by the previous study^7^. One key issue is the duration of the ^14^C input. Based on modeling, it has been proposed that a tree ring record of the event could be explained by a spike in ^14^C production that lasted less than 1 year^7^. However, owing in part to the annual resolution of the ^14^C data, they could not assess the duration in more detail^7^. *Porites* coral with an annual growth rate ≥ 10 mm/yr has now provided a high temporal-resolution ^14^C record^15^.

One 1.2-m fossil *Porites* coral, XDH, was drilled from the Xiaodonghai Reef (18°12.46′N, 109°29.93′E) from the northern South China Sea in 1997. We analysed ^14^C contents for half-annual-resolution subsamples at depths of 1.04–42.65 cm and ~2-year biweekly-resolution subsamples at depths of 12.25–17.19 cm (Fig. 1, Table S2 and S3).

## Results

**Figure 1:**
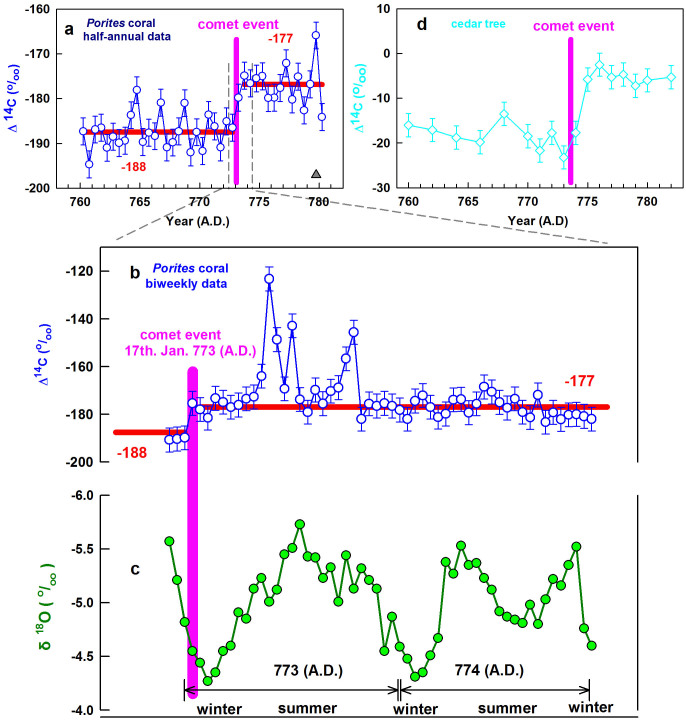
Measured radiocarbon content in coral and trees^7^. The concentration of ^14^C is expressed as Δ^14^C. For trees, Δ^14^C is the deviation (in‰) of the^14^C/^12^C ratio of a sample with respect to modern carbon (standard sample) after correcting for the age and isotopic fractionation^7^; For coral, Δ^14^C is the direct deviation (in‰) of the^14^C/^12^C ratio of a sample with respect to modern carbon after isotopic fractionation correction. (a) Half-annual and (b) Biweekly resolution record of Δ^14^C in the *Porites* coral (open blue circles with error bars) from the South China Sea (SCS) from this study. (c) Comparison of coral δ^18^O (solid green circles) [plotted to identify the seasonal cycle of sea surface temperature (SST), with the maximum seasonal coral δ^18^O value corresponding to February, the coldest month at our sample site]. (d) Annual to biennial resolution record of Δ^14^C in two cedar trees (open light blue diamonds with error bars) from Japan^7^. The vertical pink bars indicate the mysterious ^14^C increase event (M12). Japanese tree data is plotted on their original time scales while the ^230^Th age of our coral data is shifted, within quoted errors, 3.5 years young to correlate with the event. As indicted by our high-resolution coral record, this event happened in the winter, which is consistent with a big Comet event (Dai7). The thick red lines in (a) and (b) indicate the average values before and after M12. The gray triangle in (a) indicates position of ^230^Th-dated layer. Note that the δ^18^O in (c) is plotted with a reverse axis.

The ^14^C increased by ~ 15‰ in the winter of AD 773 and remained roughly constant for ~ 4 months, and then jumped up by another ~45‰ within four weeks and then dropped down in late spring, forming a spike of 45‰ high. This is followed by two smaller spikes of > 20‰ over the next 6 months until fall, and then maintained ~15‰ higher than normal values over the following several months (Fig. 1b). We obtained a ^230^Th date of AD 783 ± 14 (table S1) at a depth of 2.15 cm, which is 7 annual growth bands above the layer containing the onset of ^14^C anomalies at a depth of 16.11 cm and corresponding to an age of AD 776 ± 14. When the previously published tree ring spectrum^7^ was examined, the ^14^C content had actually started to climb in AD 773 (Fig. 1d). There are no other ^14^C increases until 200 yrs later^16^. Considering dating errors, the major ^14^C increases we observed are also likely to have occurred in AD 773 (Fig. 1a).

## Discussion

The coral ^14^C spectrum shown in Fig. 1 is difficult to be explained using normal production pathways from Galactic cosmic rays. The abrupt ^14^C increase by ~45‰ within two weeks (Fig. 1b) requires a radiation intensity 100 times stronger than the previous estimation for M12. Since the residence time of carbon dioxide in the atmosphere is 5–15 years^17^,^18^, ^14^C spikes in coral suggest highly uneven distribution. It is well established that a comet collided with the Earth's atmosphere from constellation Orion (or Shen in traditional Chinese astronomy) on 17 January AD 773, the 7th year of Emperor Dai Zong of the Tang Dynasty. The phenomenon (hereafter Dai7) lasted less than one day and had an accompanying coma that stretched across the whole sky^19^,^20^. “Dust rain” in the daytime before the “comet” implies that a considerable amount of cometary material was added to the atmosphere assuming these two events are associated. Celestial observations were especially significant to the emperors of ancient China, especially in the Tang Dynasty, and these were carefully recorded. This event was recorded in several different official archives in China^19^,^20^, included by royal celestial officers in Chang'an (now Xi'an), the capital city of the Tang dynasty (34°16′N, 108°54′E).

It is quite possible that Dai7 resulted in the M12 global abrupt ^14^C increases recorded in tree rings and corals. Comas are known to have percent levels of nitrogen by weight (in the forms of NH_3_, NH_2_, NH, etc)^21^,^22^, and are heavily exposed, as compared to nitrogen within the earth's atmosphere because of lacking a magnetic field protection^23^. Considering that meteorite usually has ^14^C and^10^Be about two orders of magnitude higher than those of rocks from the Earth's surface^24^,^25^,^26^, it is reasonable to propose that coma and comet may be expected to have ^14^C/^12^C ratios several orders of magnitude higher than that of the Earth's atmosphere^23^. Generally, ^14^C occurs in very low concentrations in the Earth's atmosphere, i.e., no more than one part per trillion of the total carbon content of the atmosphere^27^. The total amount of preindustrial ^14^C in the atmosphere was ~150 metric tonnes. Assuming an average ^14^C/^12^C ratio of 1 × 10^−6^ in the Dai7 comet, ~150 million metric tonnes of C from the Dai7 event would double the ^14^C content of the Earth's atmosphere. Assuming a C abundance of 10% in the comet, a total of ~30–150 million metric tonnes of materials would then be required to explain the ^14^C anomalies. This is only about 1–3% of the estimated total mass-loss of Haley's Comet in 1910 (ref. 28). With the considerable uncertainties surrounding the dispersal of cometary material throughout the atmosphere and shallow oceans, such a process seems commensurate with the observed ^14^C increases (Fig. 1).

**Figure 2:**
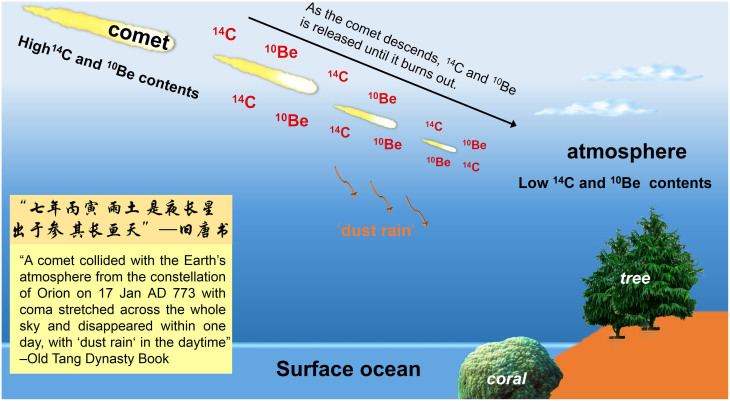
A cartoon illustrating our proposed mechanism causing a ^14^C spike--the collision of the Dai7 “Comet” with high ^14^C and ^10^Be contents with the Earth's atmosphere. As it descends, ^14^C and^10^Be is released until the comet burns out. This spike of cosmogenic ^14^C is first added to the atmosphere with its originally very low ^14^C, and the additional carbon is then incorporated into coral from the South China Sea and Japanese trees. The original record of the Dai7 “Comet” event (in Chinese with translation) is also shown in the lower left corner of the cartoon. Photos are provided by Yi Liu.

The coma ^14^C would have been dispersed into the Earth atmosphere heterogeneously (Fig. 2). Because the coma is far better exposed to cosmic radiations than the nucleus, it should have a much higher ^14^C/^12^C ratio. A considerable proportion of the coma with its higher ^14^C/^12^C content is probably scattered and absorbed into the outer atmosphere. The bulk of the cometary material with^14^C/^12^C values that are much lower than that of the coma, but still considerably higher than the Earth's atmosphere, may be expected to descend into the troposphere and become incorporated into corals and trees. Four months later, the high^14^C/^12^C material captured in the outer atmosphere (stratosphere) mixes downward into the troposphere, a process facilitated by summer storms, and is absorbed by corals, resulting in their high and fluctuating ^14^C spikes in coral (Fig. 1b). After another six months, the enriched ^14^C material becomes well mixed and imparts elevated ^14^C levels to the whole atmosphere (Fig. 1b).

Consistent with the ^14^C increase, there was a 30% increase in the decadal^10^Be flux record in Dome Fuji from AD 755 to 785 (refs. 7,16,29), which has been attributed to a burst of high energy γ-rays^12^. We were not able to obtain ^10^Be data in this study. Nevertheless,^10^Be is another cosmogenic isotope formed through spallation of nitrogen^12^,^14^N(n,p + α)^10^Be, or oxygen, which often co-varies with ^14^C. The increase in ^10^Be, can also be interpreted by the Dai7 event. The comet with abundant oxygen and nitrogen, could likewise produce high amounts of ^10^Be under exposure to cosmic radiation.

As an alternative, short radiation bursts, e.g., the merger of two magnetized neutron stars, can produce a spinning black hole and launch a relativistic energy jet as observed in short γ-ray bursts^30^ that might also explain the brief input of ^14^C and^10^Be (ref. 12). This could conceivably produce an interaction between the short γ-ray burst and the magnetic field of the Earth which might appear to be a comet. However, the γ-ray burst is fast and interacts with the entire magnetic field of the earth in seconds; therefore it is not easily explained as having “entered from the constellation of Shen (Orion)”^19^,^20^. It is also difficult to explain the ‘dust rain” beforehand, unless the dust rain was only a coincidence.

It has long been recognized that ^14^C and^10^Be in the Earth's atmosphere varied dramatically throughout the history of the Earth^5^,^16^,^31^, which has previously been solely attributed to cosmic radiations^1^,^2^,^3^,^4^,^13^. The coincidence of Dai7 and the ^14^C,^10^Be spikes in tree rings and coral suggests that comets might also contributed significant amount of ^14^C to the Earth's atmosphere episodically.

## Methods

### Coral core

A 1.2 - m long core of fossil *Porites* coral XDH was drilled from Xiaodonghai Reef in the northern South China Sea in 1997. Slabs of 7 mm in thickness, were sectioned, washed with ultrapure water, and dried for X-ray images. X-ray diffraction analysis shows our coral samples are 100% aragonite and scanning electron microscopy image indicates the absence of secondary aragonite around the coral part having the ^14^C spike. The subsamples were crushed and homogenized one by one in an agate mortar.

### Measurements

Sample XDH-2 at depth of 2.15 cm was dated by ^230^Th techniques^32^ in the High-Precision Mass Spectrometry and Environment Change Laboratory (HISPEC), at National Taiwan University, on a multi-collector inductively coupled plasma mass spectrometer (MC-ICP-MS) (Table S1).

Carbon-14 sample preparation was carried out in the State Key Laboratory of Isotope Geochemistry, Guangzhou Institute of Geochemistry. About 8–9 mg coral sample power was weighed and put in a special reaction quartz tube reacted with purified H_3_PO_4_ for more than 24 hours at room temperature after being kept continuously in a 1.0 × 10^−3^ torr vacuum system for at least 4 hours. CO_2_ from the reaction tube is purified and then transferred to a tube and graphitized^33^. The graphite samples were analyzed in the AMS laboratory at Peking University^34^, the standards used during the analysis are NIST OXI and OXII, the analytic precision for our samples are better than 3‰ and 5‰ for half-annual and biweekly samples, respectively.

δ^18^O measurements from the same biweekly subsamples were carried out using MAT-252 mass spectrometry equipped with Kiel II micro carbonate automatic sample input device at the Institute of Earth Environment, Chinese Academy of Sciences. The results are expressed in the delta (δ) notation relative to the Vienna Pee-Dee Belemnite (V-PDB) standard. The analytical error of the laboratory standard is approximately ± 0.2‰ for δ^18^O (ref. 35).

## Author Contributions

Y.L., W.D.S., Z.F.Z. and Z.C.P. designed and initiated the research. C.D.S. and K.X.L. analysed ^14^C. Y.L., C.C.S. and W.G.L. analysed ^230^Th age and O isotopes. Y.L., W.D.S., Z.F.Z. and M.X.L. plotted all the figures. X.C.S. provided information on the AD 773 Comet. W.D.S., Y.L., Z.F.Z. and M.X.L. prepared the manuscript.

## Supplementary Material

Supplementary Information Mysterious abrupt carbon-14 increase in coral contributed by a comet
