# Supplementary material for: Mysterious abrupt carbon-14 increase in coral contributed by a comet
Source: Sci Rep. 2014 Jan 16;4:3728. doi: 10.1038/srep03728 (PMC3893640; doi:10.1038/srep03728)
Supplement: Supplementary Information — Mysterious abrupt carbon-14 increase in coral contributed by a comet [file srep03728-s1.doc]

**Supplementary information**

**Mysterious abrupt carbon-14 increase in coral contributed by a comet**

Yi Liu, Zhao-feng Zhang, Zi-cheng Peng, Ming-xing Ling, Cheng-de Shen, Chuan-Chou Shen, Wei-guo Liu, Xiao-chun Sun, Kexin Liu, Weidong Sun^*^

*Correspondence to: Weidong Sun, weidongsun@gig.ac.cn

**This file includes:**

Tables S1 to S3

References

**Supplementary Tables**

**Supplementary Table S1.** U-Th isotopic compositions and ^230^Th date of coral fossil XDH-2

| Sample  No. | ^238^U  ppb | ^232^Th  ppt | δ^234^U  measured^a^ | ^230^Th/^238^U  activity^c^ | ^230^Th/^232^Th  ppm^d^ | Age (y)  uncorrected | Age (y)  corrected^c,e^ | δ^234^U_initial_  corrected^b^ |
| --- | --- | --- | --- | --- | --- | --- | --- | --- |
| XDH-2  2σ | 2379.3  ± 3.3 | 2165.6  ± 5.9 | 143.3  ± 1.9 | 0.013030  ± 0.000092 | 236.0  ± 1.8 | 1249.6  ± 9.1 | 1229.0  ± 14 | 143.8  ± 1.9 |

Chemistry (*1*) and MC-ICP-MS (*2*) analyses were performed in 2012.

Analytical errors are 2σ of the mean.

^a^ δ^234^U = ([^234^U/^238^U]_activity_ - 1)∙1000.

^b^ δ^234^U_initial_ corrected was calculated based on ^230^Th age (T), i.e., δ^234^U_initial_ = δ^234^U_measured_ *X* e^λ234∙^*^T^*, and *T* is corrected age.

^c^ ^230^Th/^238^U_activity_ = 1 - e^-λ234∙^*^T^* + (δ^234^U_measured_/1000)[λ_230_/(λ_230_-λ_234_)](1 - e^-(λ230 -λ234)^ *^T^* ), where T is the age. Decay constants are 9.1705 × 10^-6^ yr^-1^ for ^230^Th, 2.8221 × 10^-6^ yr^-1^ for ^234^U (*3*), and 1.55125 × 10^-10^ yr^-1^ for ^238^U (*4*).

^d^ The degree of detrital ^230^Th contamination is indicated by the ^230^Th/^232^Th atomic ratio instead of the activity ratio.

^e^ Age corrections were calculated using an estimated atomic ^230^Th/^232^Th ratio of 4 (±2) ppm (*5*).

**Supplementary Table S2.** Half-annual interval dataset

| Sample No. | Depth(cm) | Years (A.D.)^a^ | Years* (A.D.)^b^ | | Δ^14^C(‰) |
| --- | --- | --- | --- | --- | --- |
| XDH-1 | 1.04 | 783.75 | 780.25 | -184.1 | |
| XDH-2 | 2.15 | 783.25 | 779.75 | -165.9 | |
| XDH-3 | 2.99 | 782.75 | 779.25 | -176.8 | |
| XDH-4 | 4.20 | 782.25 | 778.75 | -182.6 | |
| XDH-5 | 5.22 | 781.75 | 778.25 | -175.1 | |
| XDH-6 | 6.53 | 781.25 | 777.75 | -180.2 | |
| XDH-7 | 7.53 | 780.75 | 777.25 | -172.1 | |
| XDH-8 | 8.72 | 780.25 | 776.75 | -177.6 | |
| XDH-9 | 9.59 | 779.75 | 776.25 | -179.8 | |
| XDH-10 | 11.10 | 779.25 | 775.75 | -179.9 | |
| XDH-11 | 12.31 | 778.75 | 775.25 | -175.0 | |
| XDH-12 | 13.27 | 778.25 | 774.75 | -175.5 | |
| XDH-13 | 14.04 | 777.75 | 774.25 | -176.6 | |
| XDH-14 | 14.98 | 777.25 | 773.75 | -174.9 | |
| XDH-15 | 16.11 | 776.75 | 773.25 | -179.8 | |
| XDH-16 | 17.04 | 776.25 | 772.75 | -186.5 | |
| XDH-17 | 18.13 | 775.75 | 772.25 | -185.1 | |
| XDH-18 | 18.98 | 775.25 | 771.75 | -190.9 | |
| XDH-19 | 19.92 | 774.75 | 771.25 | -186.2 | |
| XDH-20 | 20.97 | 774.25 | 770.75 | -183.6 | |
| XDH-21 | 21.96 | 773.75 | 770.25 | -191.7 | |
| XDH-22 | 23.12 | 773.25 | 769.75 | -187.5 | |
| XDH-23 | 24.13 | 772.75 | 769.25 | -192.0 | |
| XDH-24 | 25.15 | 772.25 | 768.75 | -181.0 | |
| XDH-25 | 26.72 | 771.75 | 768.25 | -187.3 | |
| XDH-26 | 27.86 | 771.25 | 767.75 | -189.8 | |
| XDH-27 | 28.92 | 770.75 | 767.25 | -190.9 | |
| XDH-28 | 29.82 | 770.25 | 766.75 | -180.9 | |
| XDH-29 | 30.64 | 769.75 | 766.25 | -188.4 | |
| XDH-30 | 32.03 | 769.25 | 765.75 | -187.6 | |
| XDH-31 | 33.03 | 768.75 | 765.25 | -189.7 | |
| XDH-32 | 34.04 | 768.25 | 764.75 | -178.1 | |
| XDH-33 | 35.05 | 767.75 | 764.25 | -183.7 | |
| XDH-34 | 36.05 | 767.25 | 763.75 | -189.4 | |
| XDH-35 | 37.02 | 766.75 | 763.25 | -189.9 | |
| XDH-36 | 37.94 | 766.25 | 762.75 | -188.5 | |
| XDH-37 | 38.90 | 765.75 | 762.25 | -191.0 | |
| XDH-38 | 39.65 | 765.25 | 761.75 | -186.5 | |
| XDH-39 | 40.58 | 764.75 | 761.25 | -186.9 | |
| XDH-40 | 41.54 | 764.25 | 760.75 | -194.7 | |
| XDH-41 | 42.65 | 763.75 | 760.25 | -187.3 | |

^a^ The chronology is based on ^230^Th age of XDH-2 and annual density bands of coral.

^b^ The chronology is based on 773 event of ^14^C profile and annual density bands of coral.

**Supplementary Table S3.** Biweekly interval dataset

| Sample No. | Depth(cm) | Year* (A.D.) | Δ^14^C(‰) | δ^18^O(‰) |
| --- | --- | --- | --- | --- |
| XDH-m-1 | 12.25 | 774 | -182.0 | -4.60 |
| XDH-m-2 | 12.34 | 774 | -180.8 | -4.76 |
| XDH-m-3 | 12.43 | 774 | -180.0 | -5.52 |
| XDH-m-4 | 12.52 | 774 | -180.2 | -5.35 |
| XDH-m-5 | 12.61 | 774 | -182.1 | -5.16 |
| XDH-m-6 | 12.70 | 774 | -179.2 | -5.22 |
| XDH-m-7 | 12.79 | 774 | -183.3 | -5.03 |
| XDH-m-8 | 12.88 | 774 | -171.9 | -4.8 |
| XDH-m-9 | 12.97 | 774 | -181.3 | -4.98 |
| XDH-m-10 | 13.06 | 774 | -179.1 | -4.81 |
| XDH-m-11 | 13.15 | 774 | -173.5 | -4.84 |
| XDH-m-12 | 13.24 | 774 | -177.3 | -4.87 |
| XDH-m-13 | 13.33 | 774 | -175.0 | -4.92 |
| XDH-m-14 | 13.42 | 774 | -170.6 | -5.12 |
| XDH-m-15 | 13.51 | 774 | -168.5 | -5.23 |
| XDH-m-16 | 13.60 | 774 | -175.6 | -5.37 |
| XDH-m-17 | 13.69 | 774 | -179.3 | -5.35 |
| XDH-m-18 | 13.78 | 774 | -173.7 | -5.53 |
| XDH-m-19 | 13.87 | 774 | -173.9 | -5.27 |
| XDH-m-20 | 13.96 | 774 | -179.8 | -5.38 |
| XDH-m-21 | 14.05 | 774 | -181.2 | -4.67 |
| XDH-m-22 | 14.14 | 774 | -177.0 | -4.51 |
| XDH-m-23 | 14.23 | 774 | -172.1 | -4.35 |
| XDH-m-24 | 14.32 | 774 | -174.4 | -4.31 |
| XDH-m-25 | 14.41 | 774 | -182.0 | -4.48 |
| XDH-m-26 | 14.50 | 773 | -178.2 | -4.59 |
| XDH-m-27 | 14.59 | 773 | -176.5 | -4.87 |
| XDH-m-28 | 14.67 | 773 | -175.4 | -4.55 |
| XDH-m-29 | 14.76 | 773 | -176.5 | -5.13 |
| XDH-m-30 | 14.85 | 773 | -175.6 | -5.21 |
| XDH-m-31 | 14.94 | 773 | -182.0 | -5.32 |
| XDH-m-32 | 15.03 | 773 | -145.6 | -5.13 |
| XDH-m-33 | 15.12 | 773 | -156.7 | -5.44 |
| XDH-m-34 | 15.21 | 773 | -168.8 | -5.01 |
| XDH-m-35 | 15.30 | 773 | -170.3 | -5.33 |
| XDH-m-36 | 15.39 | 773 | -175.5 | -5.23 |
| XDH-m-37 | 15.48 | 773 | -169.8 | -5.42 |
| XDH-m-38 | 15.57 | 773 | -179.1 | -5.43 |
| XDH-m-39 | 15.66 | 773 | -173.8 | -5.73 |
| XDH-m-40 | 15.75 | 773 | -142.9 | -5.51 |
| XDH-m-41 | 15.84 | 773 | -169.3 | -5.45 |
| XDH-m-42 | 15.93 | 773 | -148.7 | -5.12 |
| XDH-m-43 | 16.02 | 773 | -123.3 | -5.01 |
| XDH-m-44 | 16.11 | 773 | -164.0 | -5.23 |
| XDH-m-45 | 16.20 | 773 | -172.7 | -5.13 |
| XDH-m-46 | 16.29 | 773 | -173.5 | -4.85 |
| XDH-m-47 | 16.38 | 773 | -176.1 | -4.91 |
| XDH-m-48 | 16.47 | 773 | -177.0 | -4.60 |
| XDH-m-49 | 16.56 | 773 | -174.9 | -4.55 |
| XDH-m-50 | 16.65 | 773 | -173.3 | -4.35 |
| XDH-m-51 | 16.74 | 773 | -181.5 | -4.27 |
| XDH-m-52 | 16.83 | 773 | -178.0 | -4.44 |
| XDH-m-53 | 16.92 | 773 | -175.4 | -4.55 |
| XDH-m-54 | 17.01 | 772 | -189.8 | -4.82 |
| XDH-m-55 | 17.10 | 772 | -190.3 | -5.21 |
| XDH-m-56 | 17.19 | 772 | -190.7 | -5.57 |

**References for Supplementary Information (SI):**

1 C.-C. Shen *et al*., Variation of initial ^230^Th/^232^Th and limits of high precision U-Th dating of shallow-water corals. *Geochim. Cosmochim. Acta* **72**, 4201-4223 (2008)

2. C.-C, Shen *et al*., High-precision and high-resolution carbonate ^230^Th dating by MC-ICP-MS with SEM protocols. *Geochim. Cosmochim. Acta* **99**, 71-86 (2012).

3. C.-C. Shen *et al*., Measurement of attogram quantities of ^231^Pa in dissolved and particulate fractions of seawater by isotope dilution thermal ionization mass spectroscopy. *Anal. Chem.* **75**, 1075-1079 (2003).

4. H. Cheng *et al*., Improvements in ^230^Th dating, ^230^Th and ^234^U half-life values, and U-Th isotopic measurements by multi-collector inductively coupled plasma mass spectroscopy. *Earth Planet. Sci. Lett*. **371-372**, 82-91 (2013).

5. A. H. Jaffey, K. F. Flynn, L. E. Glendenin, W. C. Bentley, A. M. Essling, Precision measurement of half-lives and specific activities of ^235^U and ^238^U. *Phys. Rev. C* **4**, 1889–1906 (1971).

.
